# Understanding the Fundamental Basis for Biofilm Formation on Plastic Surfaces: Role of Conditioning Films

**DOI:** 10.3389/fmicb.2021.687118

**Published:** 2021-06-25

**Authors:** Geetika Bhagwat, Wayne O’Connor, Ian Grainge, Thava Palanisami

**Affiliations:** ^1^School of Environmental and Life Sciences, The University of Newcastle, Callaghan, NSW, Australia; ^2^NSW Department of Primary Industries, Port Stephens Fisheries Institute, Taylors Beach, NSW, Australia; ^3^Global Innovative Centre for Advanced Nanomaterials, School of Engineering, The University of Newcastle, Callaghan, NSW, Australia

**Keywords:** conditioning films, microbial attachment, plastic, biofilms formation, atomic force microscope

## Abstract

Conditioning films (CFs) are surface coatings formed by the adsorption of biomolecules from the surrounding environment that can modify the material-specific surface properties and precedes the attachment of microorganisms. Hence, CFs are a biologically relevant identity that could govern the behavior and fate of microplastics in the aquatic environment. In the present study, polyethylene terephthalate (PET) and polylactic acid (PLA) plastic cards were immersed in natural seawater to allow the formation of CFs. The changes in the surface roughness after 24 h were investigated by atomic force microscopy (AFM), and the surface changes were visualized by scanning electron microscopy (SEM). The global elemental composition of the conditioned surface was investigated by energy dispersive spectroscopy (EDS). Results indicated that marine conditioning of PET and PLA samples for 24 h resulted in an increase of ∼11 and 31% in the average surface roughness, respectively. SEM images revealed the attachment of coccoid-shaped bacterial cells on the conditioned surfaces, and the accumulation of salts of sodium and phosphate-containing precipitates was revealed through the EDS analysis. The results indicate that the increase in surface roughness due to conditioning is linked to a material’s hydrophilicity leading to a rapid attachment of bacteria on the surfaces. Further investigations into the CFs can unfold crucial knowledge surrounding the plastic-microbe interaction that has implications for medical, industrial, and environmental research.

## Graphical Abstract

The formation and composition of conditioning films.

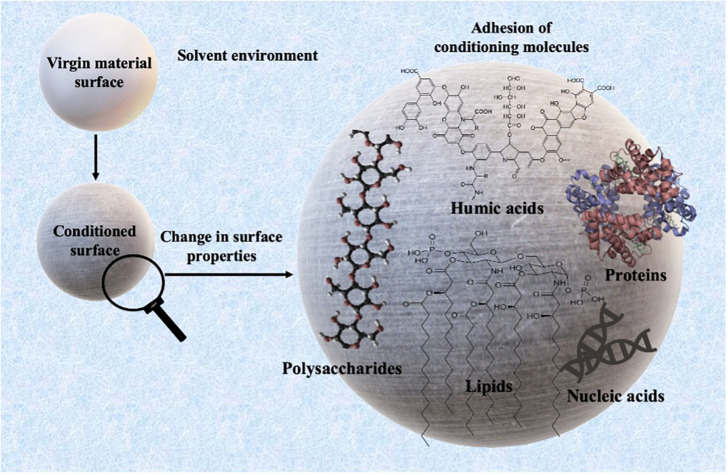

## Introduction

The global presence of plastic debris in terrestrial and aquatic environments is a serious environmental issue due to its abundance and its unidentified long-term impacts. In 2019, global plastic production reached ∼370 tons (PlasticsEurope, 2020), and it is estimated that the global annual primary plastic production may reach 1.1 billion tons in 2050 (Geyer, 2020). The total plastic waste generated so far is estimated to be 7 billion tons, out of which ∼76% is in the landfills, dumps, or in the natural environment (Geyer, 2020).

To develop plastic remediation strategies, it is necessary to understand the behavior of plastics in the environment. Upon exposure to the natural environment, physicochemical weathering (UV-induced, thermal, etc.), and microbial biofilm formation are considered to be the two fundamental processes that can influence the behavior and fate of plastics in the environment (Rummel et al., 2017; Wright et al., 2020). Microbial processes, in particular, can influence the ecological fate of plastics by governing the interactions of plastics with the biota in the natural environment (Trotter et al., 2019; Mammo et al., 2020; Rogers et al., 2020). While the harmful impacts of terrestrial (de Souza Machado et al., 2018) and aquatic plastic debris (Xu et al., 2020) on surrounding biota are frequently documented (Parthasarathy et al., 2019), the interaction of plastic with terrestrial and aquatic microorganisms is not well-characterized (Wang et al., 2021). Therefore, understanding the association of microbes and biofilm formation on plastics is crucial to understand the behavior and overall fate of plastics.

Before biofilm formation on plastics, almost immediately after the exposure of a material’s surfaces to the aqueous environment, conditioning films (CFs) are formed on the surface by molecules present in the aqueous phase (Loeb and Neihof, 1975; Rittle et al., 1990; Lawrence et al., 2016). These molecules can consist of many organic compounds such as glycoproteins (Baier, 1980), lipids, nucleic acids, ions, polysaccharides, proteins (Baier, 1980; Rittle et al., 1990; Taylor et al., 1997; Compère et al., 2001; Bakker et al., 2004), aromatic amino acids (Taylor et al., 1997), humic substances (Loeb and Neihof, 1975), absorbed carbohydrates like uronic acid, pyruvate, sulfate, proteins (Decho, 1990; Jain and Bhosle, 2009), exopolysaccharides, etc. (Berman and Passow, 2007). These biomolecules are mainly the products of the metabolic activities of aquatic organisms that include extracellular polymeric substances (EPS), exoproteome (signaling molecules), etc. Due to the complex nature of these biomolecules, the formation of CFs can potentially influence the physicochemical properties of materials. Atomic force microscope (AFM) studies have suggested that CFs can significantly alter the surface tension, charge density, and roughness (Xu and Logan, 2005; Francius et al., 2017). Furthermore, CFs may appear to modify the chemical composition of substratum surface (Hogt et al., 1985; Oga et al., 1988; Barth et al., 1989) and other physicochemical properties such as wettability and free energy, which play an important role in subsequent colonization by microorganisms (Taylor et al., 1997; Bakker et al., 2004; Bhosle, 2004; Abe et al., 2011; Hwang et al., 2013). Conditioned surfaces may also act as a chemoattractant, providing chemical stimuli to which organisms present in oligotrophic environments may actively respond by haptotaxis (directional motility up a gradient) (Lawrence et al., 2016). Therefore, CFs are considered an important prerequisite for the establishment of complex biofilms (Cooksey and Wigglesworth-Cooksey, 1995) and are regarded as a “biologically relevant entity” (Lynch et al., 2007).

**FIGURE 1:**
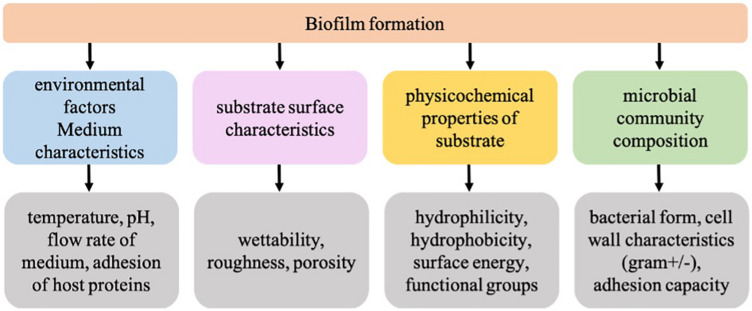
Factors influencing the formation of biofilms on a substrate.

The plastic-associated microbiota can undertake a variety of functions such as plastic degradation, xenobiotic degradation, horizontal gene transfer including transfer of antibiotic resistance or metal resistance, nitrogen fixation, quorum sensing, sulfur reduction, etc. (Bryant et al., 2016; Pinnell and Turner, 2019; Roager and Sonnenschein, 2019). Therefore, plastic-associated biofilms are the key to unlock our understanding of the behavior and fate of plastics in the environment. A recent study conducted by our group investigated the plastic microbiome of biodegradable and non-biodegradable plastic polymers and found significantly different microbiome on a biodegradable polymer (Bhagwat et al., 2021). The resulting differences in the microbiome composition could have resulted due to the inherent differences in the surface properties of biodegradable plastics such as wettability and roughness. Therefore, to understand the role of CFs in biofilm formation, it is crucial to look at surface conditioning leading to bacterial adhesion on the plastic surface as an important event governed by a variety of factors such as medium characteristics, surface properties, material properties, and microbial composition (Figure 1). In the present study subsequent changes in the surface properties of non-biodegradable polyethylene terephthalate (PET) and biodegradable poly-lactic lactic acid (PLA) plastic polymers upon a 24 h-marine conditioning were investigated. To the best of our knowledge, there is no literature on the PET and PLA marine conditioning, and it is anticipated that the results from this baseline study will add to the fundamental knowledge about the early events in biofilm formation on plastic surfaces exposed to the natural marine environment.

## Materials and Methods

### Experimental Setup for Surface Conditioning

Commercial PET and polylactic acid (PLA)-made disposable cups were chosen to conduct this study. These polymers were chosen in this study due to their frequent use in disposable consumer products such as drinking water bottles (PET) and bioplastic cups (PLA) purchased from a local supplier (MGH packaging, NSW, Australia) and cut into 2 × 2 cm cards. The cards were cleaned before the analysis by immersion in 0.1% sodium dodecyl sulfate at 40°C for 1 h, rinsing with milli-Q water (thrice), immersion in 0.1 M HCl at 40°C for 1 h, rinsing with milli-Q water (thrice), and finally drying under the nitrogen flow. Natural seawater collected from Lake Macquarie, NSW, was filtered through 11-μm filter and triplicate cards of each material type were fully immersed in 100 ml of the filtered water for 24 h at 22°C. Environmental metadata for the lake water such as temperature, pH, dissolved organic carbon, dissolved oxygen, salinity, and redox potential were measured on-site using a multiparameter water quality meter U-5000G (Horiba Ltd., Kyoto, Japan). For the chemical analyses, 200 ml of each sample was collected in sterile bottles, placed on ice, immediately transported to the laboratory, and stored at 4°C in dark. Determination of total organic carbon (TOC) in the water was carried out using a TOC analyzer (TOC-L, Shimadzu). Cards were removed after 24 h and immediately placed in a sterile box containing milli-Q water, and further analysis was conducted within 48 h.

### Atomic Force Microscopy

The changes in the nanoscale topography of the plastic samples upon conditioning were determined by atomic force microscopy (AFM). The AFM measurements of the roughness of the plastic surfaces before and after immersion in the filtered natural seawater were conducted in Milli-Q water using an Asylum MFP3D-SA AFM (Oxford Instrument, Mannheim, Germany) with Scanning Probe Image Processor (SPIPTM) v. 6.3.6 (Image Metrology A/S)^1^ as image processing and analysis software. Roughness measurements were addressed *via* AFM imaging in contact mode using silicon nitride (SiNi) cantilevers (Budget sensors, Innovative solution Bulgaria Limited, Bulgaria) with a spring constant of ∼0.06 N/m with measured inverse optical lever sensitivity of 50 nm/V. Images were acquired at a scan rate of 0.15 Hz and a scan size of 90 × 90 μm with a pixel resolution of 512 × 512. The surface topography and the average root mean square (RMS) roughness of the surfaces before and after their immersion in seawater were measured by imaging three regions (randomly selected near the centre of the cards) of 90 × 90 μm per card in Milli-Q water.

### Surface Analysis

The surface morphology of the conditioned plastic was visualized on a scanning electron microscope (SEM) (JEOL, Japan). For sample preparation, the plastics were dried under vacuum and coated with <2 nm platinum using a fine auto coater (JEC-3000FC, JEOL) and imaged at 2 kV. Following the SEM imaging, energy dispersive spectroscopy (EDS) was used at 15 kV to gauge the global elemental composition of CFs formed on the plastic surface.

## Results

### AFM Analysis

**FIGURE 2:**
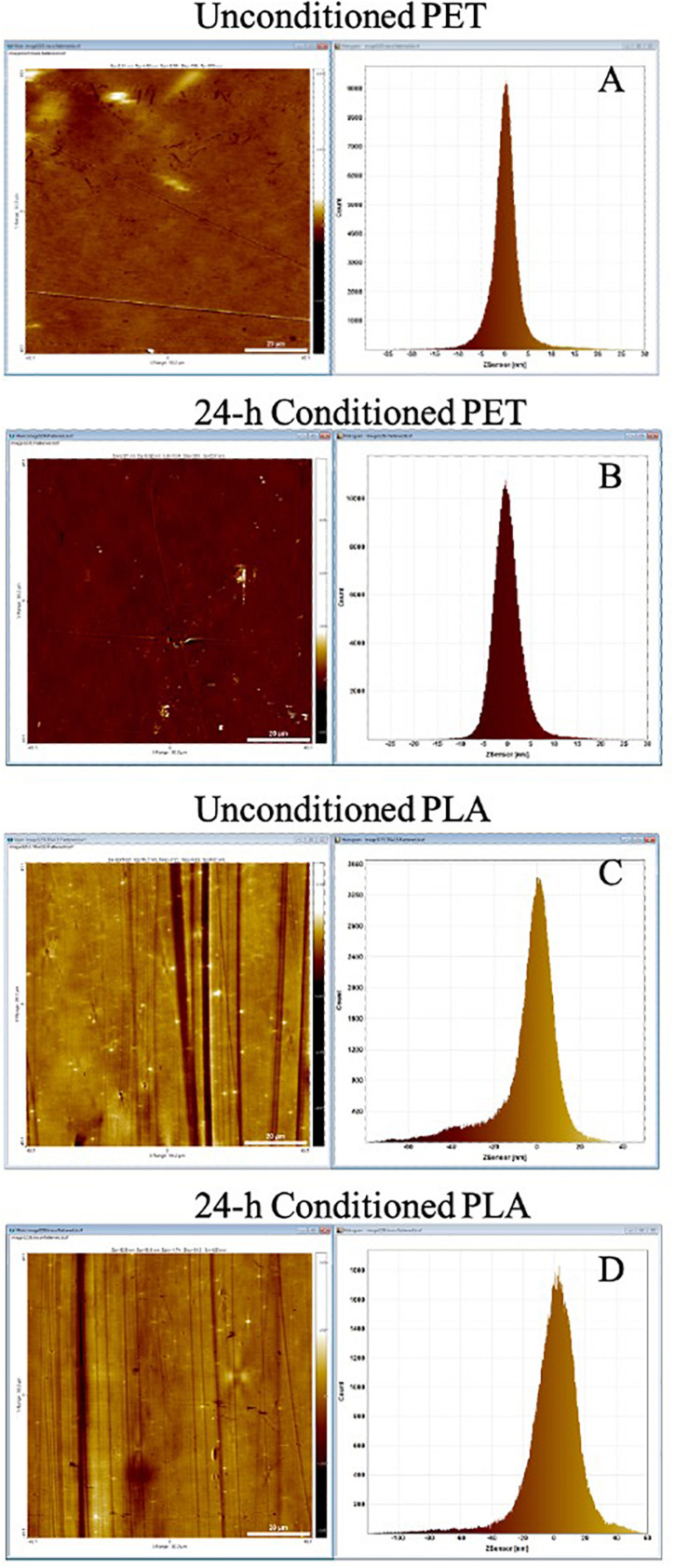
AFM topography image and pixel height histogram of the **(A)** unconditioned PET surface, **(B)** PET surface following exposure to seawater for 24 h, **(C)** unconditioned PLA surface, **(D)** PLA surface following exposure to seawater for 24 h. The standard deviation of the pixel height distribution is equivalent to the RMS roughness (*S*_*q*_) of the surface.

The lake water used for marine conditioning had a pH of 8.1 ± 0.01 and contained 14.4 ± 0.06 mg l^–1^ of TOC and 36.6 ± 0.31 g l^–1^ of total dissolved solids. The analysis of conditioned surfaces indicated an increase in the surface roughness, which was likely due to the deposition of biomolecules resulting in the formation of CFs (Figure 2). The thin lines across the surface in Figure 2 are most likely artifacts arising from the PET and PLA material’s extrusion process. The RMS roughness (Sq) of PET and PLA after 24 h immersion in seawater increased by ∼11 and 30%, respectively (Table 1). The roughness measurement parameters *S*_*a*_, *S*_*q*_, *S*_*sk*_, *S*_*ku*_, and *S*_*z*_ defined in standard ISO/DIS 25178-2 (ISO, 2012) are provided in Table 1.

**TABLE 1:** Roughness measurements calculated by SPIP^TM^ from the AFM topography images.

Polymer type		Average roughness *S*_*a*_ (nm)	Root mean squae roughness *S*_*q*_ (nm)	Surface skewness *S*_*sk*_ (nm)	Surface kurtosis *S*_*ku*_ (nm)	Peak to peak *S*_*z*_ (nm)
PET	Unconditioned	2.6	**4.9**	3.9	137.8	376.0
	24-h conditioning	2.9	**6.9**	13.4	389.7	537.2
PLA	Unconditioned	9.8	**15.7**	−2.2	9.6	506.8
	24-h conditioning	12.9	**19.6**	−1.7	10.3	425.1

*The PET and PLA cards were exposed to seawater for 24 h and the measurements were done in Milli-Q water.*

#### Adhesion Forces in Conditioned PET and PLA

**FIGURE 3:**
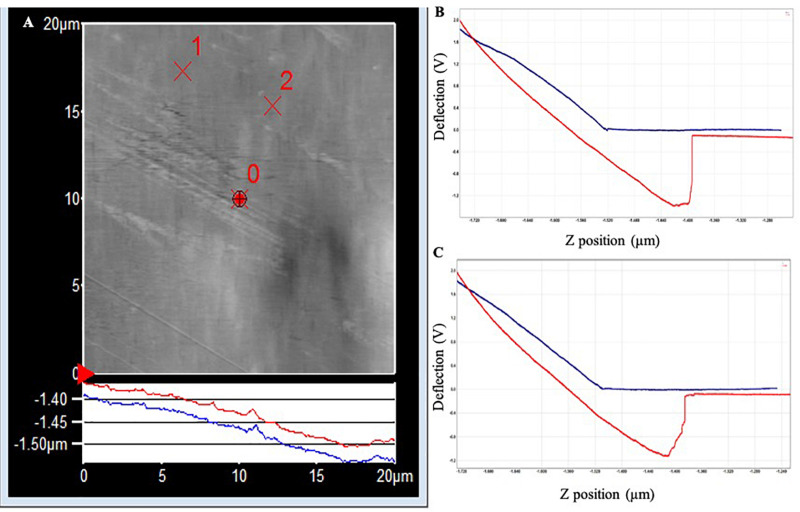
**(A)** AFM topography image of the PET surface (20 × 20 μm) following exposure to Standard Seawater for 24 h. The force curves were acquired at the locations identified as ×1 and ×2, which did not have any apparent surface abnormalities. **(B)** Force–distance curve acquired at location ×1. **(C)** Force–distance curve acquired at location ×2.

The topography image of the PET surface (Figure 3) shows a step in the red withdrawal curve, indicating the release of the adhesion force between the surface and the cantilever tip. The two withdrawal curves reach a minimum point of approximately −1.2 V on the cantilever deflection scale. The curve rises rapidly, indicating a “snapping back” of the cantilever as the adhesion force is overcome, returning to the relaxation point of the cantilever at approximately −0.075 V. An approximate adhesion force can then be calculated from the difference between these two deflection values multiplied by the optical lever sensitivity of the cantilever and the spring constant of the cantilever, i.e., approximate adhesion force = [−0.075 V – (−1.2 V)] × 50 nm/V × 0.05 nN/nm = 2.8 nN.

**FIGURE 4:**
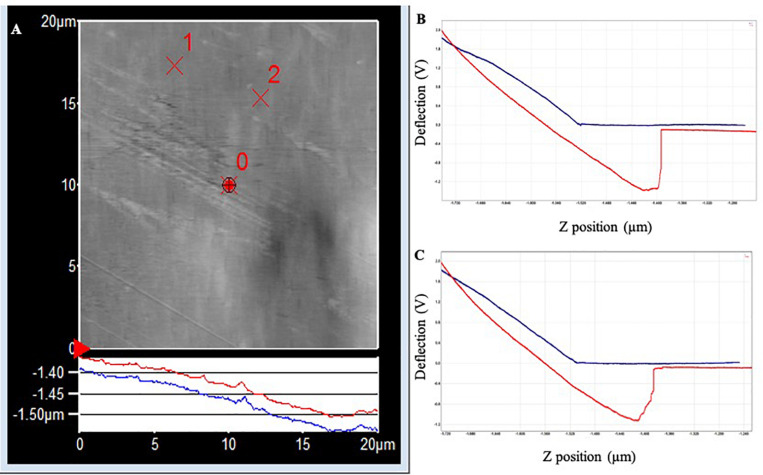
**(A)** AFM topography image (20 × 20 μm) of the PLA surface following exposure to Standard Seawater for 24 h. The force curves were acquired at the locations identified as ×1 and ×2, which did not have any apparent surface abnormalities. **(B)** Force–distance curve acquired at location ×1. **(C)** Force–distance curve acquired at location ×2.

In PLA, the two withdrawal curves reach a minimum point of approximately −2.5 V on the cantilever deflection scale (Figure 4). The curve rises rapidly, indicating a “snapping back” of the cantilever as the adhesion force is overcome, returning to the relaxation point of the cantilever at approximately 0 V. The approximate adhesion force in conditioned PLA = [0 V – (−2.5 V)] × 50 nm/V × 0.05 nN/nm = 6.3 nN.

### SEM Analysis

#### Surface Topography and Global Elemental Profiles of Conditioned Surfaces

**FIGURE 5:**
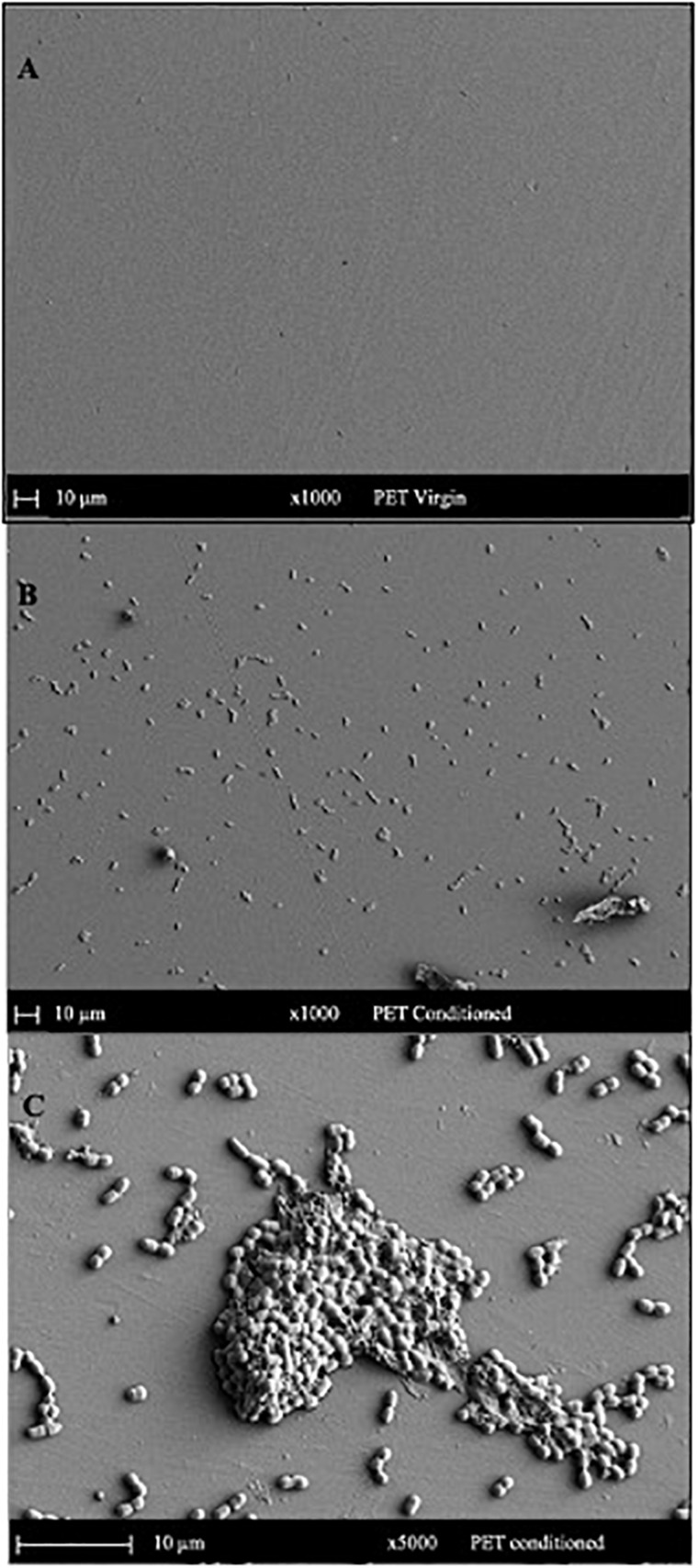
SEM images indicating the surface morphology of the **(A)** virgin PET and **(B,C)** conditioned PET cards in natural seawater. The surface of conditioned plastic shows the attachment of coccoid-shaped bacterial cells after 24 h of immersion in natural seawater at 22°C.

**FIGURE 6:**
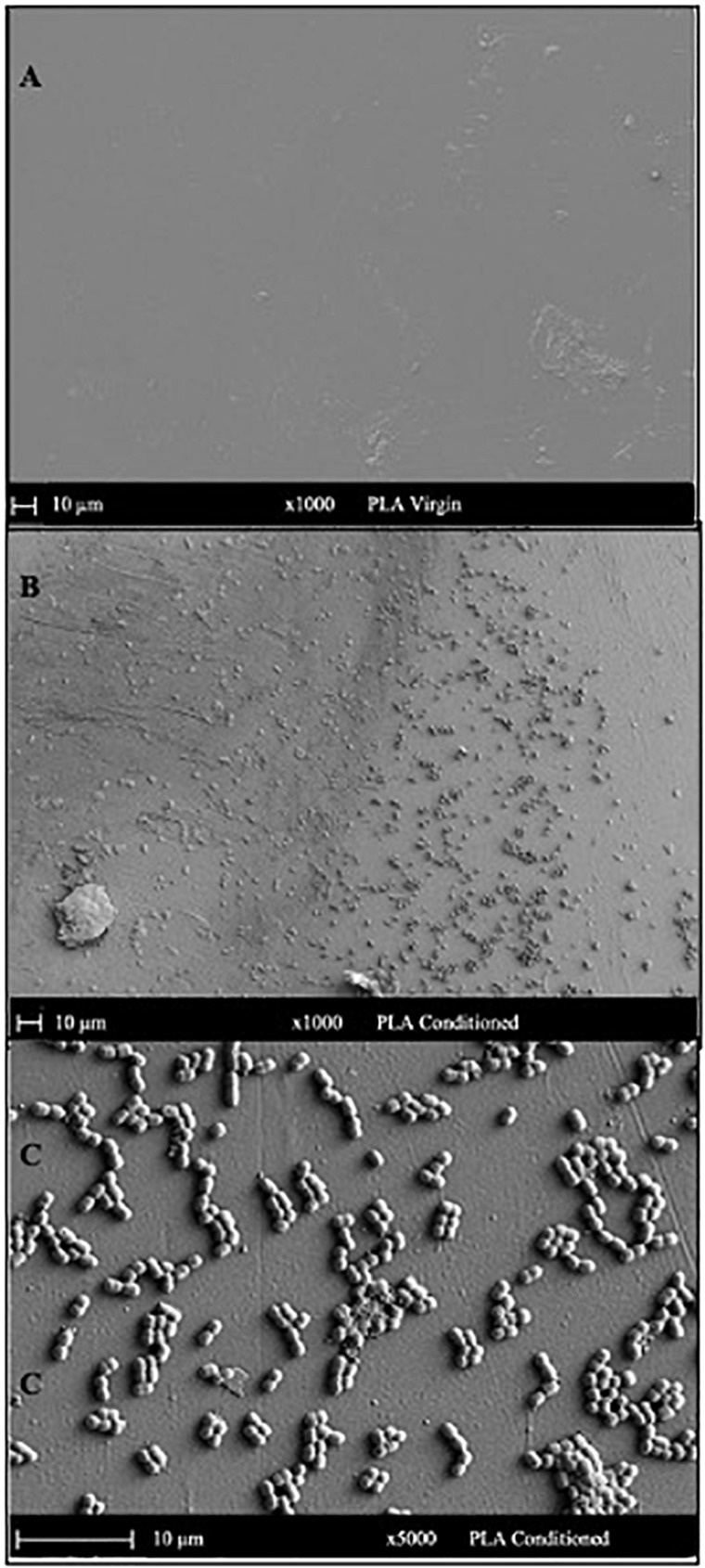
SEM images indicating the surface morphology of the **(A)** virgin PLA and **(B,C)** conditioned PLA cards in natural seawater. The surface of conditioned plastic shows the attachment of coccoid-shaped bacterial cells after 24 h of immersion in natural seawater at 22°C.

The SEM micrograph of PET (Figure 5) and PLA (Figure 6) conditioned with seawater revealed the accumulation of salt-like precipitates along with the attachment of bacterial cells from the seawater. The surface of unconditioned PET (Figure 5A) was clean compared to the conditioned PET (Figures 5B,C) and showed the attachment of coccoid-shaped bacterial cells, indicating that the surface conditioning occurs within 24 h, leading to the attachment of microbial cells as indicated in previous studies (Harrison et al., 2014). Similarly, the surface of conditioned PLA also showed the attachment of coccoid-shaped bacterial cells (Figures 6B,C). These microbial cells could be primary colonizers such as members from *Rhodobacteraceae* and *Flavobacteriaceae* family (Erni-Cassola et al., 2020), which were found to colonize different microplastics incubated in the same lake during a whole-genome analysis conducted by our research group (Bhagwat et al., 2021).

The global elemental profiles of virgin and conditioned surfaces were determined with EDS at five different points and mean values are provided in Table 2. The presence of nitrogen and phosphorus seems to increase in the conditioned samples, which could be due to the accumulation of salts of sodium and phosphate-containing precipitates (Rivadeneyra et al., 2014). The limited sensitivity of the EDS warrants the need for highly sensitive techniques to detect the compositional shift in the elemental profiles of the material surfaces upon conditioning.

**TABLE 2:** Mass percent of elements identified at the surface of virgin and conditioned PET and PLA cards submerged in natural seawater at 22°C.

Element	PET virgin	PET conditioned	PLA virgin	PLA conditioned
Carbon	75.6 ± 1.2	71.0 ± 1.4	52.2 ± 1.6	59.0 ± 2.4
Nitrogen	–	2.3	4.3	8.1
Oxygen	24.3 ± 0.5	25.6 ± 1.2	20.5 ± 1.1	23.6 ± 0.9
Sodium	–	0.3 ± 0.03	0.17 ± 0.01	0.2 ± 0.05
Magnesium	–	0.9 ± 0.07	0.97 ± 0.01	1.6 ± 0.1
Phosphorus		1.7 ± 0.4	4.2 ± 0.5	3.8 ± 0.2
Sulfur	–	–	–	0.3 ± 0.02
Calcium	–	1.1 ± 0.1	1.7 ± 0.1	2.9 ± 0.2

## Discussion

Global plastic production is rising, as is the rate at which plastics enter the environment, including the oceans (Law, 2017; Dauvergne, 2018). Currently, there is a lack of detailed data on how plastics in the ocean interact with their local environment on the micro-scale, namely, how the plastic surfaces are altered by contact with all the chemical species present in seawater, and how it can change the interaction of plastics with microbes in that environment (Geetika et al., 2021). To begin to understand the early events leading to the colonization of plastic surfaces, this study has examined the changes in the properties of two plastics, PLA and PET, after 24 h of exposure to seawater. These early changes in surface properties are referred to as CFs.

The CFs are closely linked to the fundamental concept of the “eco-corona,” which is formed when a surface comes in contact with an aquatic medium and leads to the adsorption of biomolecules such as carbohydrates, proteins, lipids, etc. on the particle surface (Fadare et al., 2020). Literature indicates that the specific and non-specific interactions mediating the attachment of microbial cells to surfaces are found to be dependent on fundamental factors such as surface hydrophobicity/hydrophilicity (van Oss, 1997; Alsteens et al., 2007), functional groups like hydroxyl (R-OH), carbonyl (R′-C=OR), and carboxyl (R=O-OH) found in different molecules (Schamberger and Gardella, 1994; Wolfe et al., 2003; Lorite et al., 2011) and surface roughness (Lorite et al., 2011; Hwang et al., 2013; Heffernan et al., 2014; Francius et al., 2017). In the present study, the higher adhesion forces calculated with AFM in PLA indicated a more hydrophilic surface. This suggests that the hydrophilic nature of plastic material is likely to encourage the accumulation of biomolecules from the surrounding environment, leading to an increase in surface roughness. Therefore, an indirect conclusion can be drawn that, compared to conditioned PET (more hydrophobic), conditioned PLA (more hydrophilic) would have a higher surface roughness that would favor rapid attachment of microbes. Similar results were found in a study in which the hydrophilic adhesion forces on conditioned polyamide 6 (comparatively hydrophilic) were found to be higher than on conditioned homopolymer made of vinylidene fluoropolymer (inherently hydrophobic) (Francius et al., 2017).

Contrastingly, studies have shown a strong correlation of initial bacterial settlement with surface hydrophobicity, surface wettability, and molecular flexibility (Sanni et al., 2015; Nauendorf et al., 2016). The sensitivity of biomaterials to microbial adherence, investigated in several laboratories, followed the order latex > Silicone > PVC > Teflon > polyurethane > stainless steel > Titanium (Weinstein and Darouiche, 2001; Pascual, 2002; Green, 2006). These studies indicate that the hydrophobic PET used in the present study may be preferred by microbes for attachment over PLA despite the comparatively slower increase in the surface roughness of PET over a short duration due to CFs. However, these previous studies were all carried out on medical devices in a human setting, as opposed to the current study on plastics in an environmental setting. The polymers that form CFs and the range of microbes that are present would be expected to be very different between these settings. Furthermore, compared to hydrophilic substrates, the accumulation of conditioning biomolecules may be slower on hydrophobic materials, which may influence the rate of microbial attachment and biofilm formation. In addition, the complex and diverse composition of plastic polymers including the intentionally added additives could further affect the composition of the plastic microbiome (Restrepo-Flórez et al., 2014).

The interplay between polymer surface properties, the adherence of conditioning biomolecules, and microbial attachment is clearly complex and multifactorial. What is clear from this study, though, is that it happens rapidly. Hydrophilic surfaces accumulate biomolecules that increase surface roughness more rapidly than hydrophobic ones, which may favor microbial attachment. Hydrophobic surfaces showed less change in surface properties but may be naturally more amenable to microbial attachment. Subsequently, both surfaces showed significant colonization by bacteria in 24 h.

## Implications of CF Research

The attachment and growth of microorganisms on conditioned surfaces is the norm rather than the exception and has important implications for research. It is well-established that surface conditioning is the first step of microbial attachment that leads to biofilm formation. Despite decades of study, the fundamental understanding of physicochemical and biotic factors controlling microbial colonization is still limited and makes it hard to effectively manage the process. Furthermore, the research on CFs is still in its infancy mainly because the characterization of CFs requires sensitive analytical techniques appropriate to the task along with intensive sample handling and manipulation methodologies to avoid contamination. Therefore, it is crucial to understand and characterize the CF components, identify the factors controlling the surface properties, and improve techniques for characterizing these primary films on a variety of plastic polymers with different chemical makeup.

Biofilm formation of plastic materials can have both positive and negative implications. For instance, in medical settings, biofilm formation is generally undesirable, and therefore, the fundamental knowledge about the biofilm–plastic interaction can further be developed for creating biofilm-resistant materials for medical applications (Jain and Bhosle, 2009; Yuan et al., 2017). Furthermore, this knowledge can be extremely useful in designing anti-fouling surfaces for marine industries (Cao et al., 2011). The negative impacts of plastic-associated biofilms can also be surrounding the accumulation of pathogens and transport of antibiotic, metal, etc. resistance genes (Jacquin et al., 2019; Bowley et al., 2020). Contrastingly, biofilm formation is desirable on the plastic surface to attract bacteria that can degrade plastic and associated environmental contaminants such as hydrocarbons and other xenobiotic compounds (Roager and Sonnenschein, 2019). Therefore, this knowledge can be instrumental in creating quickly compostable plastic materials that encourage the attachment of microbes on the surface for faster degradation. Further investigations on the dynamics of the early changes on the plastic surfaces lead by CFs are needed to understand the biomolecular interactions that precede biofilms formation which influences the long-term behavior and fate of plastics in the environment.

## Data Availability Statement

The raw data supporting the conclusions of this article will be made available by the authors, without undue reservation.

## Author Contributions

GB carried out the experiments, conceptualized the work, analyzed the data, and composed the manuscript. WO’C and IG provided critical feedback and assisted with writing and preparation of the manuscript. TP provided funding and critical feedback. All authors read and approved the final manuscript.

## Conflict of Interest

The authors declare that the research was conducted in the absence of any commercial or financial relationships that could be construed as a potential conflict of interest.

## Footnotes

1 www.imagemet.com
